# Supplementary material for: The benefits of higher LMR for early threatened abortion: A retrospective cohort study
Source: PLoS One. 2020 Apr 20;15(4):e0231642. doi: 10.1371/journal.pone.0231642 (PMC7170252; doi:10.1371/journal.pone.0231642)
Supplement: Attachment — Submitted filename: response to reviewers.docx [file pone.0231642.s002.docx]

Dear Editor,

We have studied the valuable comments from reviewers carefully, and tried our best to revise the manuscript.The point to point responds to the reviewer’s comments are listed as following:

Responds to the reviewer’s comments:

**Reviewer 1**

**Comment 1:** Although the authors clarified the meaning of abbreviation, some are not a good candidate for the abbreviation. For example, TA might not be well accepted in the clinical use. In the introduction, TA as an initial word might not be appropriate.

**Response:** After referencing a lot of literature, I found that no one use TA as an abbreviation for threatened abortion. In order to be well accepted in the clinical use, I have changed “TA” to “threatened abortion”.

**Reviewer 2**

**Comment 1:** The use of simple hematological parameters to predict the various kinds of the diseases has been evaluated in the many studies. Nearly almost studies favored their clinical value. It is hard for me to believe it, although the aforementioned data have supported it. Therefore, some data had better be provided in the Table 1. Absolute number of white cells, absolute number of each subpopulation of white cells, such as neutrophil, lymphocyte, monocyte, eosinophils and/or others should be provided.

Response: After referencing a lot of literature, I found that no one use TA as an abbreviation for threatened abortion. In order to be well accepted in the clinical use, I have changed “TA” to “threatened abortion”.

**Response:** The absolute number of white blood cells, neutrophil, lymphocyte, monocyte and eosinophils, platelet has been replenished in the table 1. Details are in the following table:

| WBC, medium (IQR), 10^9^/L | 7.98 (6.63-9.50) |
| --- | --- |
| Eosinophils, medium (IQR), 10^9^/L | 0.11 (0.07-0.17) |
| Neutrophil, medium (IQR), 10^9^/L | 5.25 (4.04-6.49) |
| Lymphocyte, medium (IQR), 10^9^/L | 1.98 (1.66-2.39) |
| Monocyte, medium (IQR), 10^9^/L | 0.47 (0.38-0.55) |
| Platelet, medium (IQR), 10^9^/L | 238.00 (206.00-270.00) |
